# KLF10 inhibits cell growth by regulating PTTG1 in multiple myeloma under the regulation of microRNA-106b-5p

**DOI:** 10.7150/ijbs.45999

**Published:** 2020-05-15

**Authors:** Mimi Zhou, Jinqiu Chen, Hui Zhang, Hailing Liu, Huan Yao, Xiaman Wang, Wanggang Zhang, Yingren Zhao, Nan Yang

**Affiliations:** 1 Department of Infectious Diseases, the First Affiliated Hospital of Xi'an Jiaotong University, Yanta West Road No. 277, Xi'an 710061, China; 2 Department of Hematology, the Second Affiliated Hospital of Xi'an Jiaotong University, West Five Road No. 157, Xi'an 710004, China

**Keywords:** KLF10, multiply myeloma, PTTG1, miR-106b-5p, proliferation

## Abstract

Krüppel-like factor 10 (KLF10) has been identified as an important regulator in carcinogenesis and cancer progression. However, the role of KLF10 in multiply myeloma (MM) development and progression remains unknown. In present study, we found that KLF10 mRNA and protein were down-regulated in MM tissues and cell lines. Notably, KLF10 inhibited cell proliferation, cell cycle progression and promoted apoptosis *in vitro* and *in vivo*. Furthermore, we confirmed that KLF10 inhibited β-catenin nuclear translocation and inhibited PTTG1 transcription. PTTG1 knockdown could mimic the biological effects of KLF10. Moreover, we demonstrated that KLF10 expression was regulated by miR-106b-5p. In MM tissues, miR-106b-5p has an inverse correlation with KLF10 expression. Conclusively, our results demonstrated that KLF10 functions as a tumor suppressor in regulating tumor growth of MM under regulation of miR-106b-5p, supporting its potential therapeutic target for MM.

## Introduction

Multiply myeloma (MM) is a plasmablast malignancy characterized by heterogenetic plasma cells (PC) clonal proliferation in the bone marrow 1. It is the second most common hematological cancer worldwide and accounts for 20% of all deaths from hematological malignancy 2. Despite the survival is largely improved in recent year, due to the development of the novel chemo-therapies, like proteasome inhibitor and immunomodulator, combining with autologous stem cell transplant, majority of patients become refractory to treatment and ultimately relapse, which makes MM remain an incurable disease 3. Therefore, the precise molecular mechanisms of MM pathogenesis still need to be elucidated for innovative therapeutic strategies.

Krüppel-like factor 10 (KLF10), also called TGFβ inducible early gene-1 (TIEG1), is classified as a member of the Krüppel-like family of transcription factors 4. By binding to Sp-1-GC rich DNA sequences, KLF10 regulates gene transcription and impacts multiple pathways of the physiological and pathological processes, including bone metabolism 5, cardiac hypertrophy 6, neovascularization 7, T cell differentiation 8,9, as well as tumorigenesis. In multiple neoplasm cells, KLF10 was found to be deregulated involving proliferation, apoptosis, and cell cycle arrest. KLF10 expression in breast carcinomas was displayed less than one-half of levels in normal breast epithelium and highly associated with tumor stages 10. Notably, overexpression of KLF10 exerted anti-proliferative effects and induced apoptosis in pancreatic cancer 11,12. Moreover, mRNA expression profiling revealed LSAMP suppressed osteosarcomas proliferation possibly through upregulation of KLF10 13. Therefore, KLF10 has been suggested to be a tumorigenesis suppressor. However, the functional role of KLF10 and its transcriptional regulation mechanism in MM has not been elucidated yet.

In the present study, we demonstrated that KLF10 expression was significantly down-regulated in human clinical MM and cell lines. We confirmed that KLF10 overexpression regulated cell proliferation, cycle and apoptosis of MM cells by affecting β-catenin signaling pathway. Moreover, we demonstrated that KLF10 suppressed PTTG1 transcription, which plays critical role in MM development. In addition, we identified KLF10 expression was regulated by miR-106b-5p in MM cells. Taken together, our findings suggest that KLF10 acts as a tumor suppressor in MM progression.

## Materials and Methods

### Samples and cell lines

Primary plasma cells purified by CD138 immunomagnetic bead (Miltenyi Biotec, Auburn, CA) from bone marrow were obtained from 38 newly diagnosed MM patients and five healthy donors. CD138 flow cytometry determined that plasma cell purity was routinely ≥90%. This research study was approved by the Ethical Committee of Second Affiliated Hospital of Xi'an Jiaotong University and all patients provided the informed consents.

Human MM cell lines (RPMI8226, NCI-H929, and U266) were purchased from China Center for Type Culture Collection (Beijing, China) and cultured in RPMI 1640 media (Gibco, Grand Island, NY, USA) with 10% heat-inactivated foetal bovine serum (Gibco), penicillin (100 U/mL), and streptomycin (100 μg/mL). HEK293T were grown in DMEM media (Gibco) with the same supplement. All cells were maintained at 5% CO_2_ and 37 °C temperature in the incubator.

### Lentivirus transduction and oligonucleotide transfection

Lentiviruses encoding KLF10 (LV-KLF10) and control (LV-control) were purchased from Obio (Shanghai, China). RPMI8226 and U266 were infected with lentiviruses at MOI of 100 in the presence of 5 μg/mL Polybrene (Obio). siRNA against PTTG1 (si-PTTG1), non-targeting scrambled siRNA (si-Control), miR-106b-5p inhibitor (anti-miR-106b-5p), and inhibitor negative control (anti-miR-NC) were synthesized by Sangon Biotech (Shanghai, China). The transfection was performed as described before 14.

### RNA extraction and quantitative real-time PCR analysis

Total RNA was extracted from cells using Trizol reagent (Invitrogen). Total RNA was respectively reverse-transcribed using the PrimeScript RT reagent kit (Takara, Dalian, China) for mRNA and miRNA First-Strand Synthesis kit (Takara) for miRNA. Real-time PCR was performed using SYBR Green RT-PCR kit (Takara). Gene expression was measured in triplicate and data were processed using 2^-ΔΔCT^ method and normalized to GAPDH or U6 as the internal control. Primers of KLF10 (HQP018084), PTTG1 (HQP061080), GAPDH (HQP064347), miR-106b-5p (HmiRQP0029), and U6 (HmiRQP9001) were purchased from GeneCopoeia (Guangzhou, China).

### Protein extraction and Western blot analysis

Total protein was extracted by RIPA buffer supplemented with protease phosphatase inhibitors (Roche, Basel, Switzerland). Cytoplasmic and nuclear protein was extracted by the Nuclear and Cytoplasmic Extraction kit (Thermo Fisher). Western blot analysis was conducted as described previously 14.

### Proliferation, apoptosis, and cell cycle assay

Cell proliferation rates were evaluated using a CCK8 assay at 0, 24, 48, and 72 h post-transduction. Apoptosis and cell cycle were determined by flow cytometry at indicated time points. The detailed experiment was performed as previous reported 14.

### Luciferase reporter assay

To illustrate the relationship between KLF10 and PTTG1, wild-type (wt) (CTCACGCCCGT) or mutant (mt) promoters of PTTG1 were inserted into pGL3 vectors and co-transfected with plasmids containing KLF10 or NC into HEK293T by Lipofectamine 2000 Reagent (Thermo Fisher Scientific). Cells were harvested at 48 h after the transfection and measured by Dual Luciferase Reporter Assay System (Promega Corporation, Fitchburg, WI, USA). Luciferase activities were expressed as the luminescence of Firefly relative to Renilla. To identify the association between miR-106b-5p and KLF10, HEK293T was co-transfected with wt or mt 3'UTR of KLF10 and either miR-106b-5p mimic or miR-control and analyzed by the same system.

### Xenograft mouse model

Eight 4-6-week-old female BALB/c nude mice (Center of Laboratory Animals, Health and Science Center of Xi'an Jiaotong University, Xi'an, China) were randomized into two groups. KLF10 or NC stably-overexpressed RPMI8226 cells (1×10^7^) were injected subcutaneously with 150 μL Matrigel basement membrane matrix (Becton Dickinson) into the mice of each group. Then the diameters were measured every 3 days and tumor volume was calculated as previously described 14. After 3 weeks, the mice were sacrificed by cervical dislocation under anesthesia with ether and the xenograft tumor tissue was fixed in formalin. Animal experiment protocols were approved by the Animal Committee of Xi'an Jiaotong University.

### Statistical analysis

Statistical analysis was performed by GraphPad Prism 5 software. All data represent the mean ± standard deviation (SD), and results were analyzed using the Mann-Whitney U test. *p* < 0.05 was considered statistically significant.

## Result

### KLF10 was downregulated in MM primary cells and cell lines

**Figure 1:**
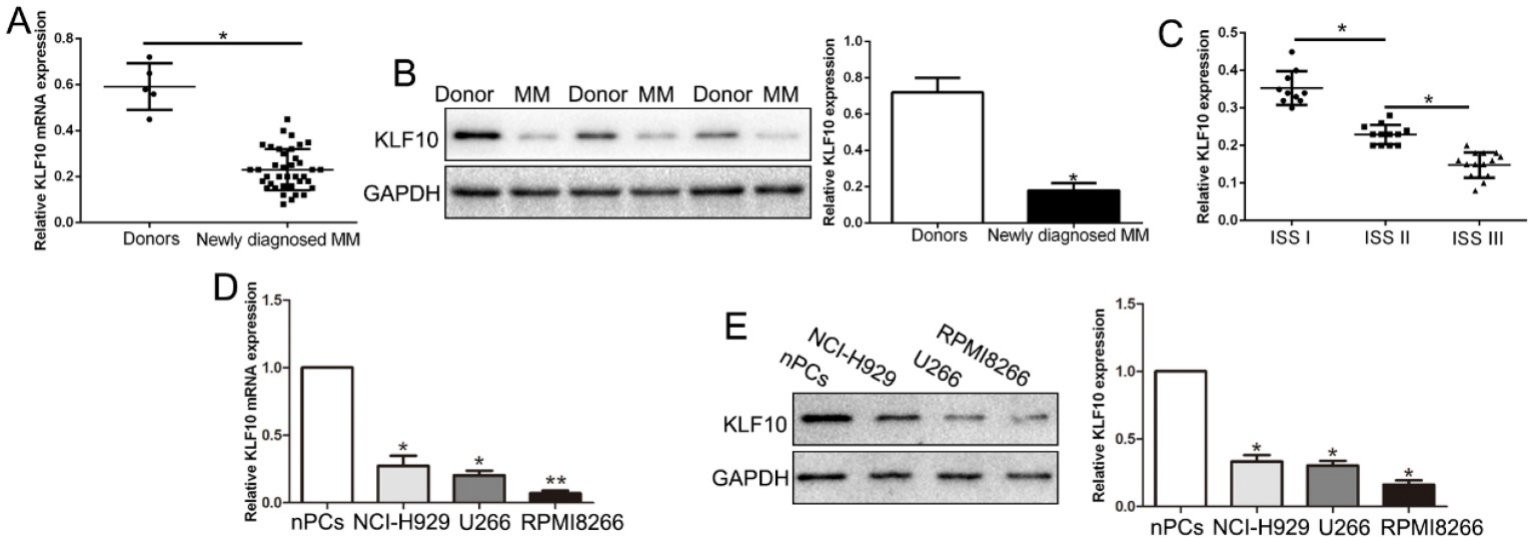
** KLF10 is significantly down-regulated in MM tissues and cell lines. (A)** Relative KLF10 mRNA expression levels in MM and healthy donors were determined by qRT-PCR. **(B)** Representative Western blot analysis of KLF10 expression in the MM and healthy donors was shown. **(C)** KLF10 level was compared between MM tissues of different ISS stage. The expression of KLF10 mRNA **(D)** and protein **(E)** in three MM cell lines was significantly decreased compared to that in the nPCs cells. n = three repeats with similar results. *P < 0.05 by ANOVA. **P < 0.01.

We first evaluated the expression of KLF10 in bone marrow derived from 38 MM patients and ten health donors. qRT-PCR and Western blot analysis exhibited that KLF10 mRNA and protein was significantly decreased in MM patients compared with health controls (Figure 1A-B, respectively, P < 0.05). The GEO dataset (GSE6477) from R2: Genomics Analysis and Visualization Platform (http://r2.amc.nl) consistently showed down-regulated KLF10 in MM (Figure S1, P = 0.0052). Moreover, the expression of KLF10 was associated with ISS stage. The data showed that KLF10 was decreased in advanced stage (Figure 1C, P < 0.05). We further validated the expression of KLF10 in three MM cell lines (RPMI8226, NCI-H929, and U266) and normal plasma cells (nPCs). Similarly, the expressions of KLF10 mRNA and protein were notably downregulated in MM cell lines in comparison with nPCs (Figure 1D-E, respectively, P < 0.05). RPMI8226 and U266 expressing relatively low levels of KLF10 were selected for further studies.

### KLF10 promoted apoptosis and inhibits proliferation and cell cycle transition *in vitro*

**Figure 2:**
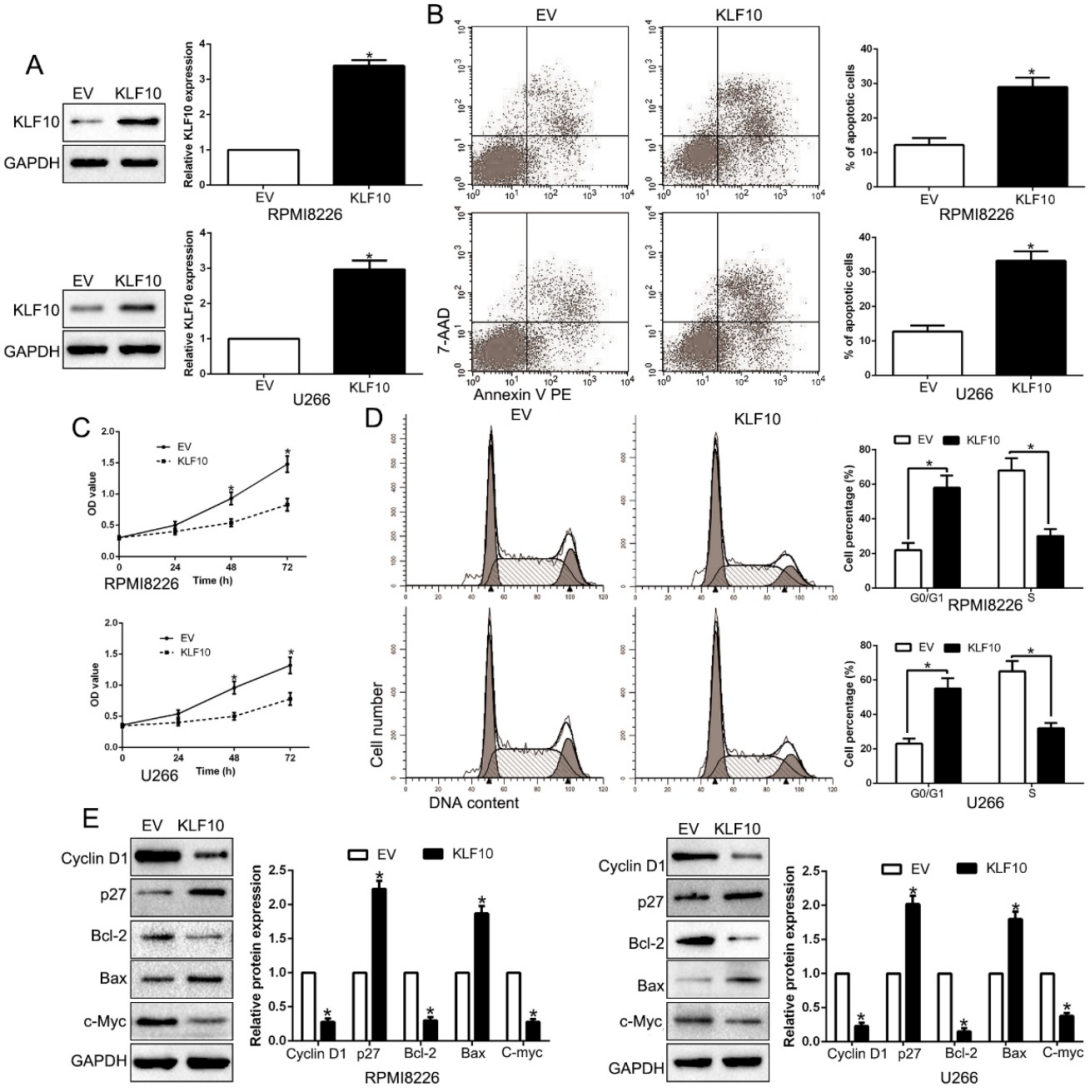
** KLF10 inhibits cell proliferation, cell-cycle progression and promotes apoptosis in MM cell. (A)** RPMI8226 and U266 cells that were transduced with corresponding KLF10 overexpression vectors were subjected to WB for KLF10. **(B)** Flow cytometry checked the effects of KLF10 up-regulation on apoptosis. **(C)** KLF10 overexpression inhibited cell proliferation in RPMI8226 and U266 cells. **(D)** Effects of KLF10 overexpression on the cell cycle progression of MM cells were measured by flow cytometric analysis. **(E)** WB measured the cycle- and apoptosis-associated factors. n = six independent experiments. *P<0.05.

To investigate possible biological function of KLF10, KLF10-overexpressed MM cell lines (RPMI8226 and U266) were established by lentivirus transduction and confirmed by Western blot (Figure 2A, P < 0.05). A remarkable increase of the proportion of apoptotic cells was observed in MM cell lines stably expressing KLF10 compared with control cells (Figure 2B, P < 0.05). Moreover, upregulation of KLF10 triggered significant inhibition of cell proliferation in time-dependent manner in both RPMI8226 and U266 (Figure 2C, P < 0.05). In addition, the flow cytometry testified that KLF10 overexpression resulted in more cells arrest in G0/G1 phase and fewer in S phase than NC (Figure 2D, P < 0.05). Compared with NC-transduced cells, the KLF10-transduced cells expressed less Cyclin D1, Bcl-2, and c-Myc proteins but more p27 and Bax proteins (Figure 2E, P < 0.05). Furthermore, KLF10 knockdown showed opposite effects on NCI-H929 cells (Figure S2). Taken together, these results indicate that enforced expression of KLF10 inhibits survival and growth of MM cells.

### KLF10 regulated tumor growth *in vivo*

**Figure 3:**
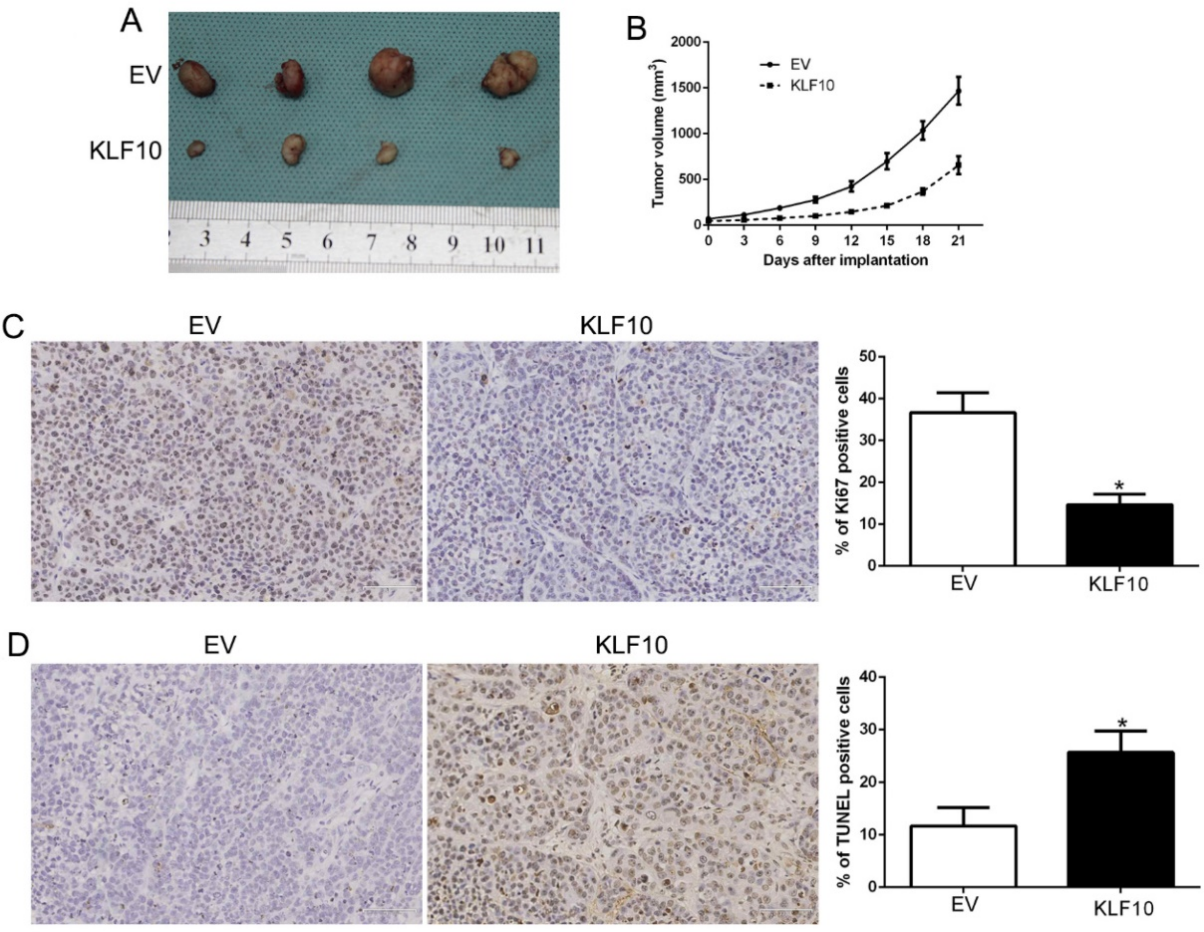
** KLF10 inhibits tumor growth and promotes apoptosis *in vivo*. (A)** Representative pictures of MM xenografts from RPMI8226-KLF10 and RPMI8226-control. **(B)** Tumor growth curve revealed that KLF10 overexpression significantly inhibited tumor growth *in vivo*. Tumor nodules were subjected to immunohistochemical staining for Ki-67 **(C)** and TUNEL **(D)** assays and quantitative analysis. Representative immunostaining and TUNEL assays revealed that KLF10 overexpression significantly decreased the number of Ki-67 positive cells and increased the number of apoptotic cells. *P<0.05.

Finally, we assessed the anti-MM activity of KLF10 in nude mice bearing subcutaneous RPMI8226 xenografts. There was a significant reduction in tumor formation in mice injected with KLF10-overexpressed MM cells when compared with NC (Figure 3A-B, P < 0.05). We also stain the plasma cell marker CD138 (Figure S3). Additionally, IHC analysis of excised tumors confirmed that elevated KLF10 notably suppressed the expression of Ki-67 protein, which indicated the proliferation activity of MM cells (Figure 3C, P < 0.05). Besides, KLF10-overexpressing tumors displayed a significantly higher apoptotic index, as indicated by the number of TUNEL-positive cells (Figure 3D, P < 0.05). Together, these results demonstrated potent anti-MM activity of KLF10 *in vivo*.

### KLF10 suppressed the activation of Wnt signaling

**Figure 4:**
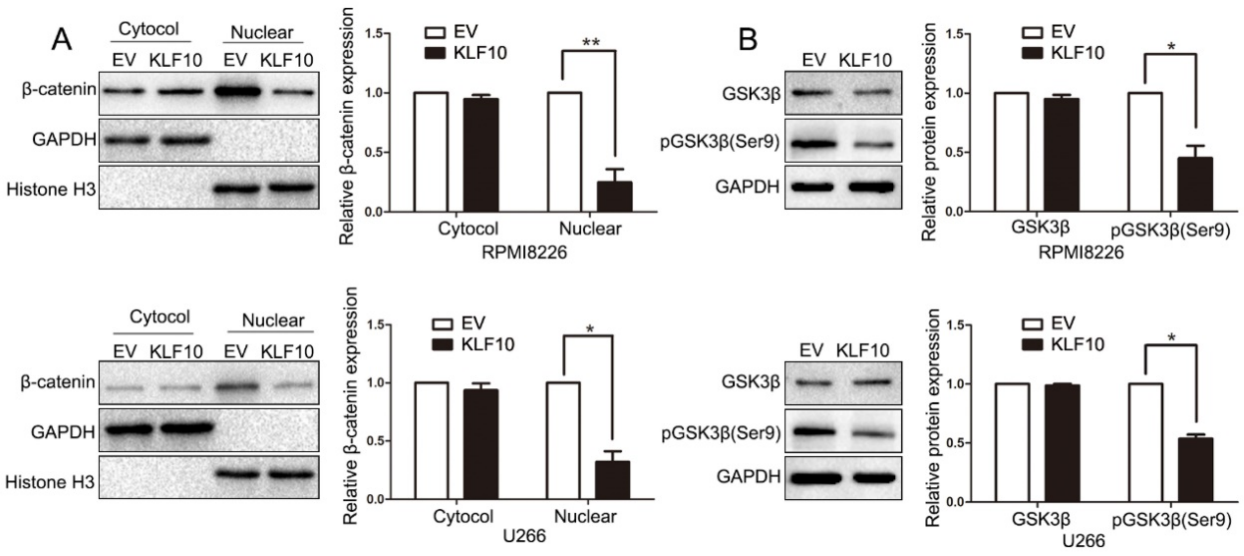
** KLF10 inhibits activation of Wnt signaling. (A)** WB showed that KLF10 overexpression suppressed β-catenin nuclear accumulation in RPMI8226 and U266 cells. **(B)** WB revealed that KLF10 overexpression suppressed GSK3β phosphorylation in MM cells. n = six independent experiments. *P<0.05, **P<0.01.

The unrestrained growth of tumor cells is generally attributed to activation of essential pathways. Active Wnt signals are identified as a hallmark in MM tumorigenesis 15-17. In the above results, we found that KLF10 downregulated the expression of Cyclin D1 and c-Myc, which were known as Wnt-downstream targets. Thus, we next investigated whether Wnt pathway was involved in KLF10-induced changes in MM. Although the cytosolic accumulation remained same, the accumulation of β-catenin in the nucleus was largely declined in KLF10-transduced cells (Figure 4A, P < 0.05). In addition, the enhanced KLF10 remarkably repressed the phosphorylation of GSK3β on Ser-9 in comparison with NC (Figure 4B, P < 0.05). Similarly, we confirmed that the activation of Wnt signaling was suppressed by KLF10 in subcutaneous RPMI8226 xenografts (Figure S4, P < 0.05). Therefore, we demonstrated that Wnt signaling was involved in the KLF10-induced anti-MM function.

### PTTG1 was the direct target of KLF10 in MM

**Figure 5:**
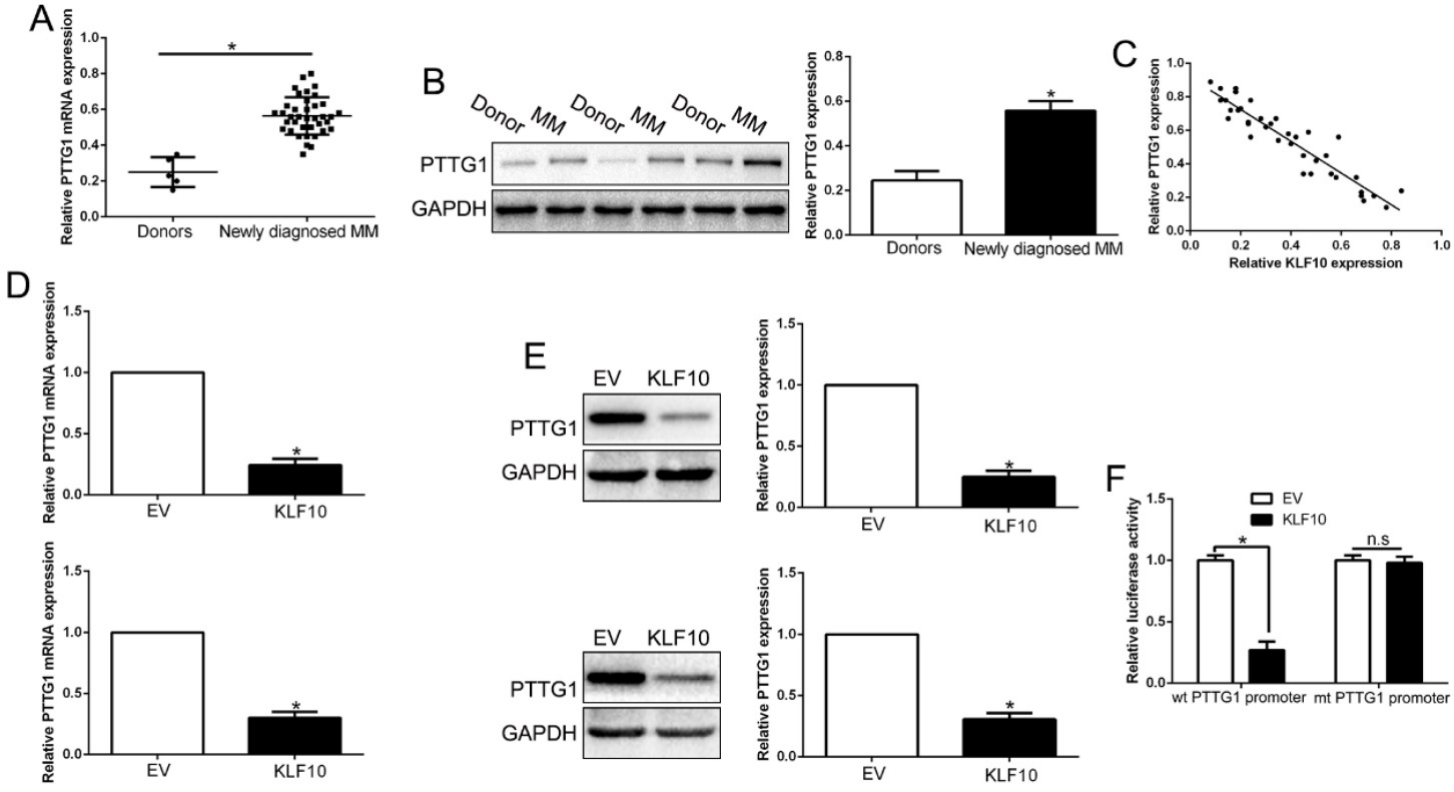
** KLF10 inhibited PTTG1 transcription in MM cells. (A)** Relative PTTG1 mRNA expression levels in MM and healthy donors were determined by qRT-PCR. **(B)** Representative Western blot analysis of PTTG1 expression in the MM and healthy donors was shown.** (C)** A significant inverse correlation between the KLF10 and PTTG1 was observed in MM tissues. KLF10 overexpression inhibited PTTG1 mRNA **(D)** and protein **(E)** expression in MM cells. **(F)** Luciferase reporter assays confirmed that KLF10 inhibited PTTG1 transcription in HEK293 cells. n = three repeats with similar results. *P<0.05, n.s: no significance.

Well known as a transcription factors, KLF10 was involved in the regulation of a variety of genes in different tissues 18-20. PTTG1 was a potential target of KLF10 in cardiac hypertrophy 6,21 but their relationship in MM remains unclear. To investigate the association between KLF10 and PTTG1, we first analyzed the expression of PTTG1 in primary MM samples. Both mRNA and protein level of PTTG1 were significantly elevated in MM patients compared with health donors (Figure 5A-B, P<0.05). Interestingly, the expression of PTTG1 protein displayed an inverse correlation to KLF10 protein (r=-0.8146, Figure 5C, P < 0.05). The GEO dataset (GSE6401) from R2: Genomics Analysis and Visualization Platform (http://r2.amc.nl) showed an inverse relationship between KLF10 and PTTG1 (Figure S5, P = 0.02). We further analyzed the effect of KLF10 on the expression of PTTG1. KLF10 largely inhibited the mRNA and protein level of PTTG1 in comparison with NC (Figure 5D-E, P<0.05). Finally, to validate KLF10-dependent regulation of PTTG1, we cloned the PTTG1 promoter into an expression vector downstream of the luciferase reporter gene, which was co-transfected into HEK293T cells together with KLF10 or NC. Overexpression of KLF10 induced a remarkable suppression of the luciferase activity in presence of wt promoter but no noticeable changes in presence of mt one (Figure 5F, P < 0.05). These results demonstrated that KLF10 directly downregulated PTTG1 through binding to its promoter.

### Inhibition of PTTG1 mimics KLF10-induced biological effects on MM

**Figure 6:**
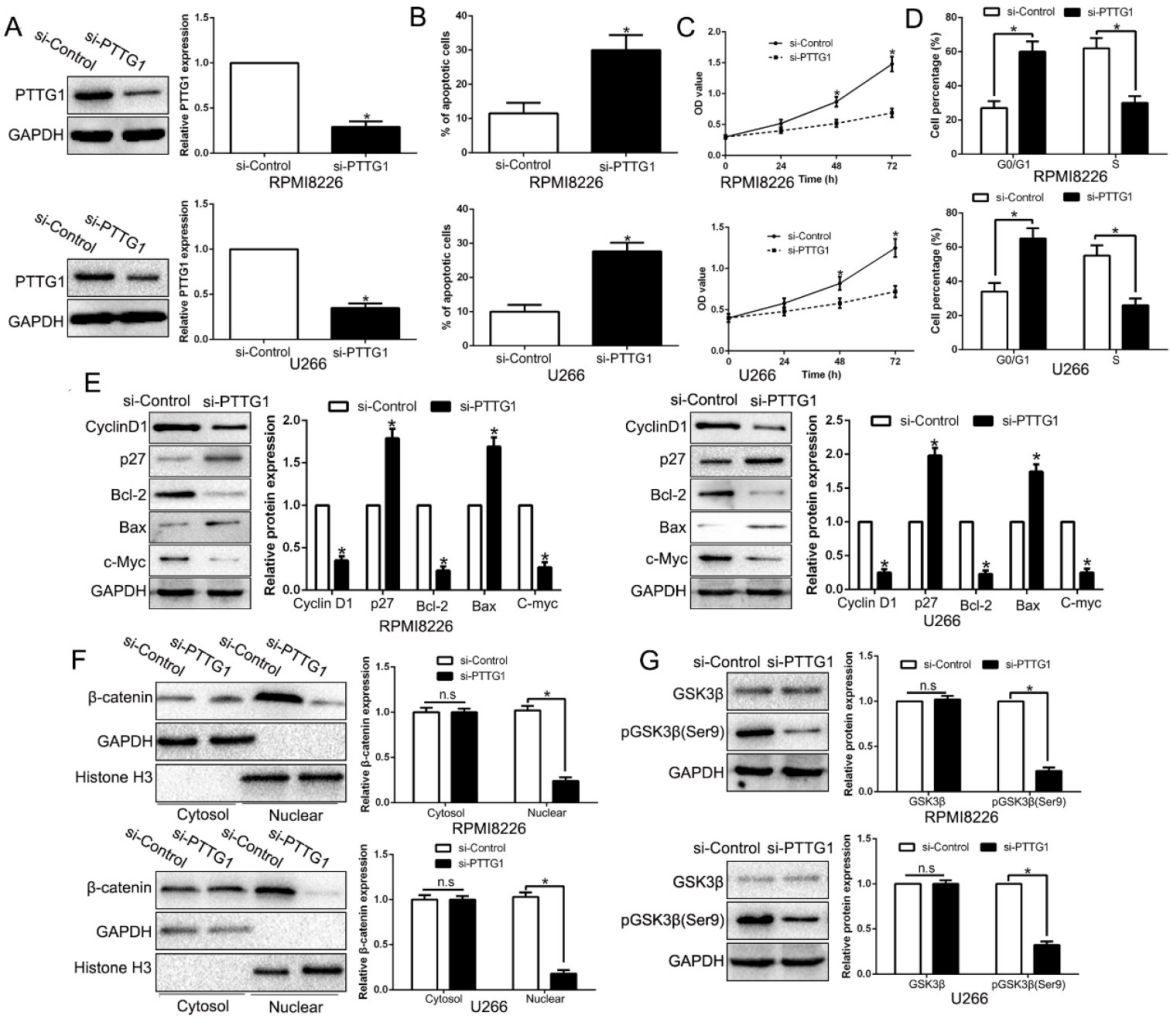
** Inhibition of PTTG1 mimics KLF10-induced biological effects on MM. (A)** WB showed the effects of PTTG1 siRNA to knockdown PTTG1. PTTG1 knockdown promoted apoptosis **(B)** and inhibited cell proliferation **(C)** and cell cycle progression **(D)**. **(E)** WB measured the cycle- and apoptosis-associated factors. **(F)** WB showed that PTTG1 knockdown suppressed β-catenin nuclear accumulation in MM cells. **(G)** WB revealed that PTTG1 knockdown suppressed GSK3β phosphorylation in MM cells. n = six repeats with similar results. *P<0.05, n.s=no significance.

To examine the function of PTTG1, we first constructed the PTTG1-knockdown MM cells (Figure 6A, P < 0.05). As expected, the downregulation of PTTG1 promoted the apoptosis, inhibited the proliferation, and induced the cycle arrest at G0/G1 phase in MM cells by regulating the related factors (Figure 6B-E, P < 0.05). Furthermore, anti-PTTG1 resulted in β-catenin degradation and the dephosphorylation of GSK3β (Figure 6F-G, P < 0.05). Thus, downregulation of PTTG1 could mimic the KLF10-induced the biological effects on MM.

### MiR-106b-5p directly downregulated KLF10 in MM

**Figure 7:**
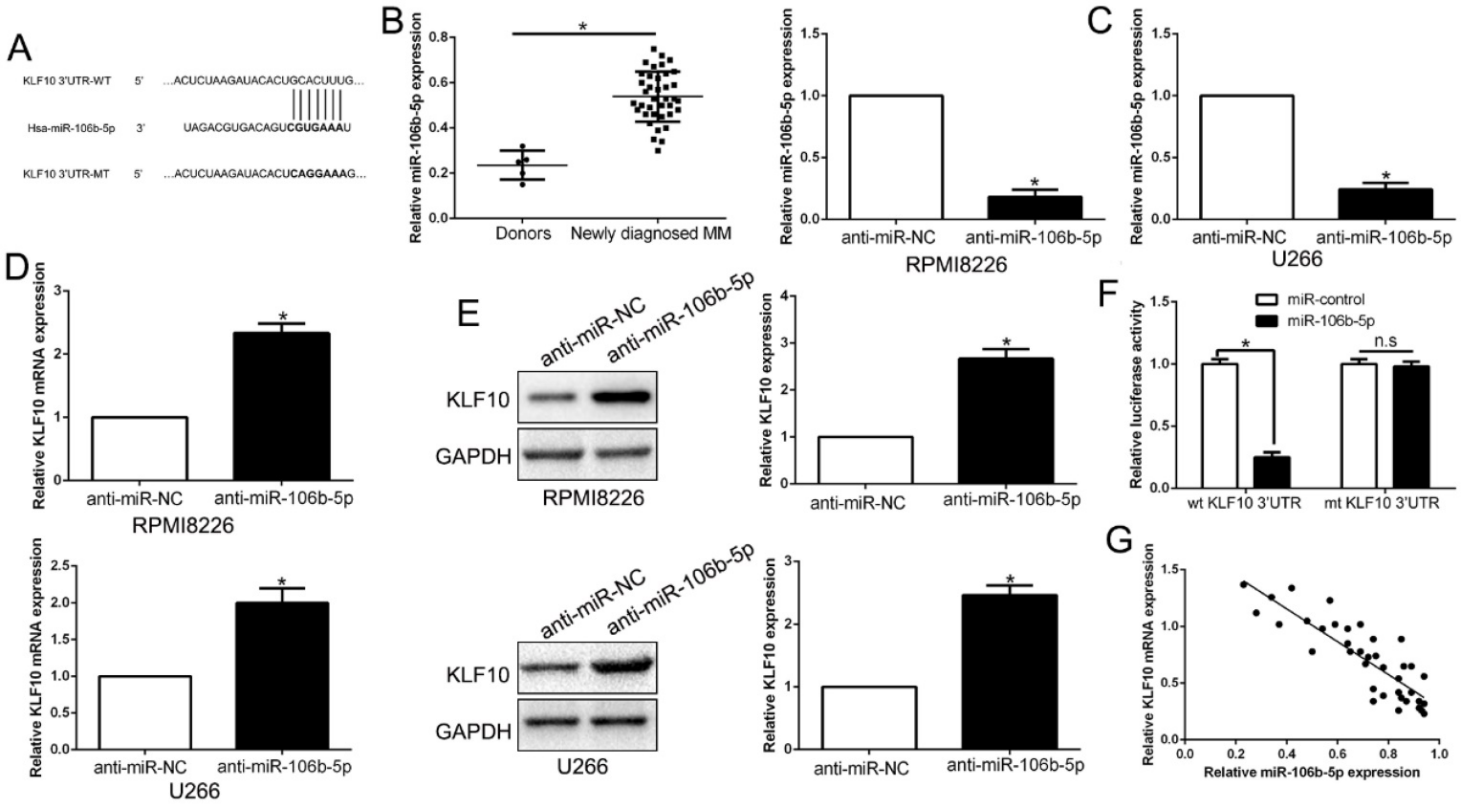
** KLF10 is identified as a direct target of miR-106b-5p in MM. (A)** miR-106b-5p and its putative binding sequence in the 3'-UTR of KLF10. The mutant binding site was generated in the complementary site for the seed region of miR-106b-5p. **(B)** miR-106b-5p was up-regulated in MM tissues compared to healthy donors. **(C)** miR-106-5p was knockdown by the miR-106b-5p inhibitors and measured by qRT-PCR. **(D)** qRT-PCR analysis of KLF10 mRNA expression in MM cells with anti-miR-106b-5p or anti-miR-NC vector transfection. **(E)** miR-106-5p knockdown increased the expression of KLF10 protein in MM cells. **(F)** miR-106b-5p significantly suppresses the luciferase activity that carried wild-type (wt) but not mutant (mt) 3'-UTR of KLF10. **(G)** A significant inverse correlation between the mRNA levels of KLF10 and miR-106b-5p was observed in MM tissues. n = six repeats with similar results. *P<0.05, n.s=no significance.

It is well documented that miRNA translationally represses gene mRNA production by binding to the seed sequence in the 3'-UTR of the target RNA in various hematological malignancies 22. Therefore, we interrogated TargetScan and miRanda to identify the controlling miRNA of KLF10. With conserved target sites and good context score percentile/mirSVR score, miR-106b-5p was predicted as a candidate target in either of two bioinformatics databases (Figure 7A). Moreover, miR-106b-5p was up-regulated in MM compared to healthy donors (Figure 7B, P < 0.05). To elucidate the association of miR-106b-5p and KLF10, miR-106b-5p knockdown in MM cell lines was conducted (Figure 7C, P < 0.05). qRT-PCR and Western blot showed that the downregulation of miR-106b-5p promoted the expression of KLF10 at both mRNA and protein levels (Figure 7D-E, P < 0.05). The luciferase reporter assay demonstrated that miR-106b-5p markedly decreases the luciferase activity of wt KLF10 3'UTR, but not that of mt KLF10 3'UTR (Figure 7F, P < 0.05). In addition, we confirmed an inverse correlation between miR-106b-5p and KLF10 expression in MM tissues (Figure 7G, r=-0.7891, P < 0.05). We also found that the downregulation of miR-106b-5p could mimic the KLF10-induced the biological effects on MM (Figure S6, P<0.05). These data strengthen the role of miR-106b-5p as KLF10 negative regulator in MM.

## Discussion

KLF10 encodes a three-zinc-finger Krüppel-like transcription factor, which is originally cloned from human osteoblasts (OBs) as a primary response gene following TGF-β treatment 23. KLF10 mimics TGF-β action and plays a role in the development of human osteosarcoma 13, pancreatic carcinoma 24, breast cancer 25 and renal cancer 26. KLF10 deficient mice showed more increased cell proliferation in skin and earlier onset of skin cancers than control littermates 27. KLF10 expression was significantly upregulated by homoharringtonine and Velcade and KLF10 was a key regulator which can induce and promote apoptosis through the mitochondrial apoptotic pathway 23. KLF10 protein expression was tightly associated with cell cycle-dependent events and multiple cell cycle-regulatory molecules 28. Here, we investigated the activity and mechanism of KLF10 in inhibiting MM cells growth and survival.

In the present study, we found that KLF10 expression was nearly absent in MM primary samples and cell lines. To address the biological effects of KLF10 in MM, we demonstrated that KLF10 inhibited MM cell proliferation, cell cycle progression and promoted apoptosis by regulating cycle- and apoptosis- associated factors* in vitro*. Moreover, we showed that KLF10 suppressed tumor growth and the Ki67 and TUNEL staining confirmed that KLF10 inhibited cell proliferation and promoted apoptosis in *vivo*. We also confirmed that KLF10 inhibited β-catenin nuclear transition and suppressed Wnt pathway. KLF10 inhibited PTTG1 expression by regulating its transcription. PTTG1 knockdown mimics the biological effects of KLF10 *in vitro*. Increasing evidence confirmed that abnormal miRNAs have been verified to be crucial regulators in the initiation and progression of human cancer. We searched the bioinformation database and showed that miR-106b-5p could bind to KLF10 3'UTR. Luciferase reporter assays showed miR-106b-5p could directly bind to the 3'UTR of KLF10 and regulated KLF10 expression. Moreover, miR-106b-5p negatively regulated the expression of KLF10 mRNA and protein in MM cells. In MM tissues, miR-106b-5p showed an inverse correlation with KLF10 expression. These data further confirm that KLF10 down-regulation was caused by miR-106b-5p overexpression. Conclusion, these results suggest that KLF10 was a downstream target of miR-106b-5p in MM.

We demonstrated that KLF10 was down-regulated in MM tissues and cell lines. We confirm that KLF10 inhibits cell proliferation, cell cycle progression and promotes apoptosis *in vitro* and *in vivo*. KLF10 inhibited β-catenin nuclear translocation. KLF10 inhibited PTTG1 expression by regulating its transcription. Additionally, we determined that miR-106b-5p regulated KLF10 expression in MM cells. These findings support the potential role of KLF10 as an attractive therapeutic target for MM treatment.

## Supplementary Material

Supplementary figures and tables.
