# KLF10 inhibits cell growth by regulating PTTG1 in multiple myeloma under the regulation of microRNA-106b-5p: Erratum

**DOI:** 10.7150/ijbs.77067

**Published:** 2022-08-20

**Authors:** Mimi Zhou, Jinqiu Chen, Hui Zhang, Hailing Liu, Huan Yao, Xiaman Wang, Wanggang Zhang, Yingren Zhao, Nan Yang

**Affiliations:** 1 Department of Infectious Diseases, the First Affiliated Hospital of Xi'an Jiaotong University, Yanta West Road No. 277, Xi'an 710061, China; 2 Department of Hematology, the Second Affiliated Hospital of Xi'an Jiaotong University, West Five Road No. 157, Xi'an 710004, China

In our paper, the authors noted one error in Fig. 3A. The subcutaneous xenograft for tumor formation picture was misused. The authors carefully checked the original data and found that the images were unintentionally included from another project (ongoing in the same lab) due to the similarity of appearance and file name. All the original pictures from this experiment were taken on the same day. We confirmed that this mistake in Figure 3A did not affect the research results and conclusion of this article. All authors have agreed to the Erratum, and we apologize for the negligence in our work and hope to get the opportunity to correct this mistake. Figure 3A should be corrected as follows.

## Figures and Tables

**Figure 3:**
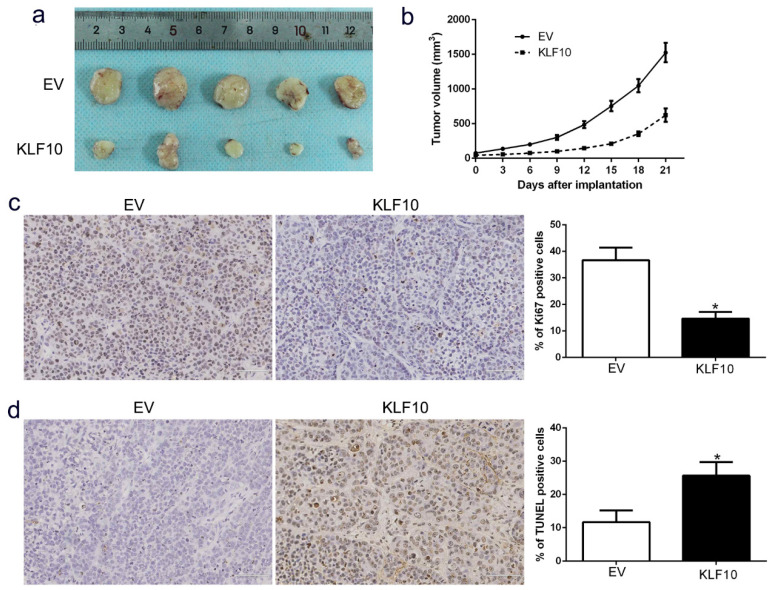
** KLF10 inhibits tumor growth and promotes apoptosis in vivo.** (A) Representative pictures of MM xenografts from RPMI8226-KLF10 and RPMI8226-control. (B) Tumor growth curve revealed that KLF10 overexpression significantly inhibited tumor growth in vivo. Tumor nodules were subjected to immunohistochemical staining for Ki-67 (C) and TUNEL (D) assays and quantitative analysis. Representative immunostaining and TUNEL assays revealed that KLF10 overexpression significantly decreased the number of Ki-67 positive cells and increased the number of apoptotic cells. *P<0.05.

