# Supplementary material for: KLF10 inhibits cell growth by regulating PTTG1 in multiple myeloma under the regulation of microRNA-106b-5p
Source: Int J Biol Sci. 2020 May 15;16(12):2063–71. doi: 10.7150/ijbs.45999 (PMC7294933; doi:10.7150/ijbs.45999)
Supplement: Supplementary file 1 — Supplementary figures and tables. [file ijbsv16p2063s1.pdf]

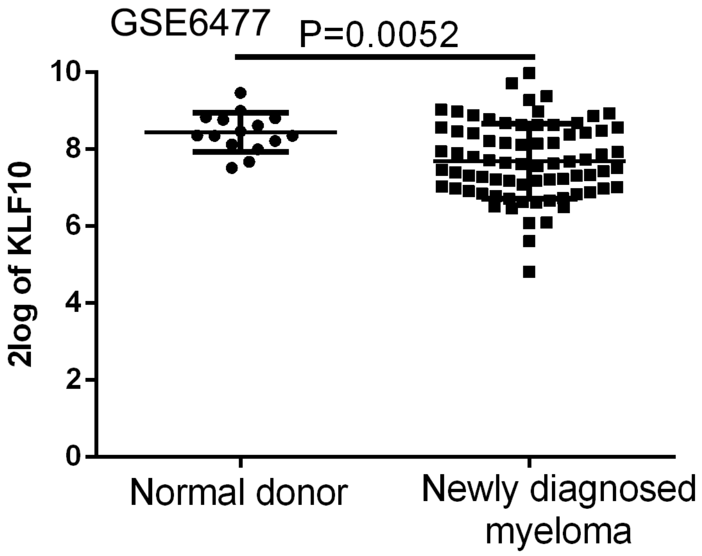

Figure S1 The GEO dataset (GSE6477) from R2: Genomics Analysis and Visualization Platform (http://r2.amc.nl) consistently showed down-regulated KLF10 in MM.

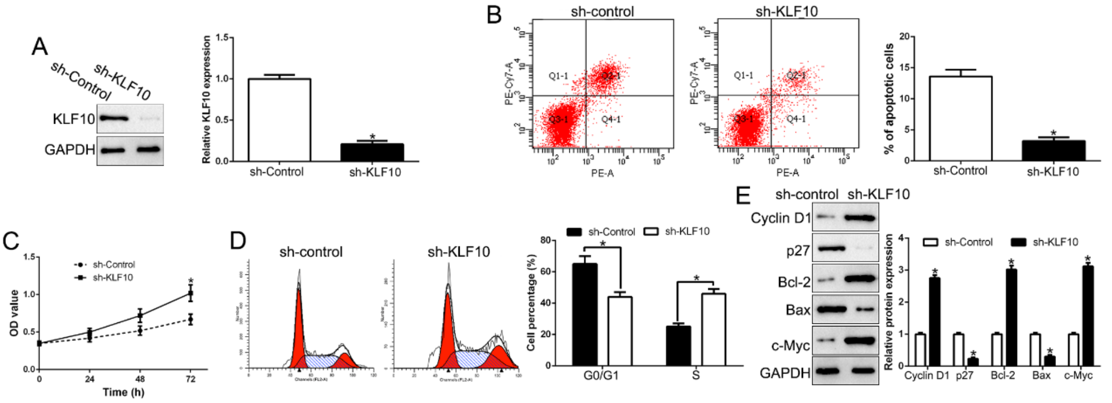

Figure S2. KLF10 knockdown promoted cell proliferation, cell-cycle progression and inhibited apoptosis in NCI-H929 cell. (A) NCI-H929 cells that were transduced with corresponding KLF10 shRNA vectors were subjected to WB for KLF10. (B) Flow cytometry checked the effects of KLF10 knockdown on apoptosis. (C) KLF10 knockdown promoted cell proliferation in NCI-H929 cells. (D) Effects of KLF10 knockdown on the cell cycle progression of NCI-H929 cells were measured by flow cytometric analysis. (E) WB measured the cycle- and apoptosis-associated factors. n = six independent experiments. *P<0.05.

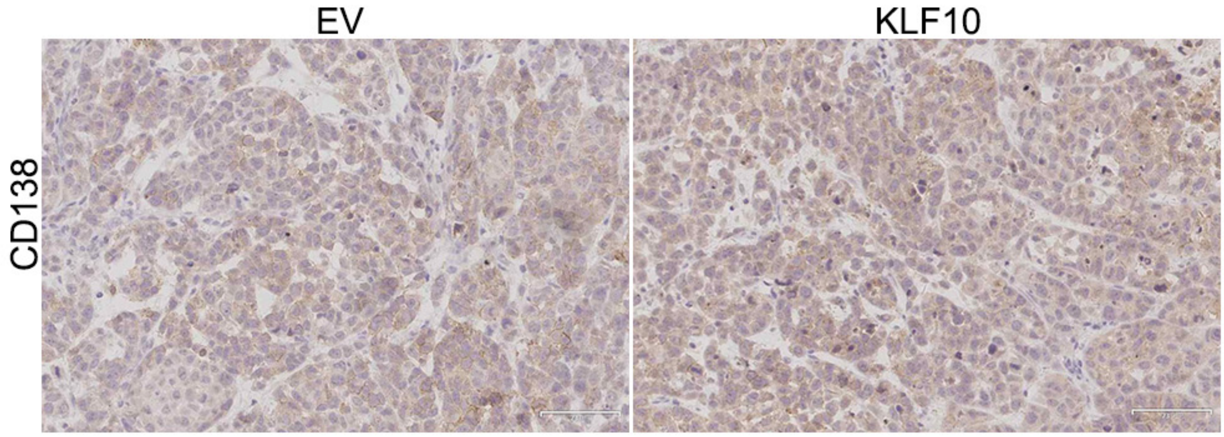

Figure S3. Tumor nodules were subjected to immunohistochemical staining for the plasma cell marker CD138.

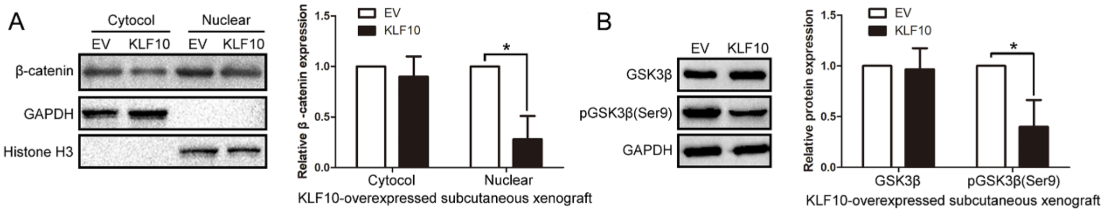

Figure S4. KLF10 suppressed activation of Wnt signaling *in vivo*. (A) WB showed that KLF10 overexpression suppressed β-catenin nuclear accumulation in subcutaneous RPMI8226 xenografts. (B) WB revealed that KLF10 overexpression suppressed GSK3β phosphorylation in subcutaneous RPMI8226 xenografts. *P<0.05.

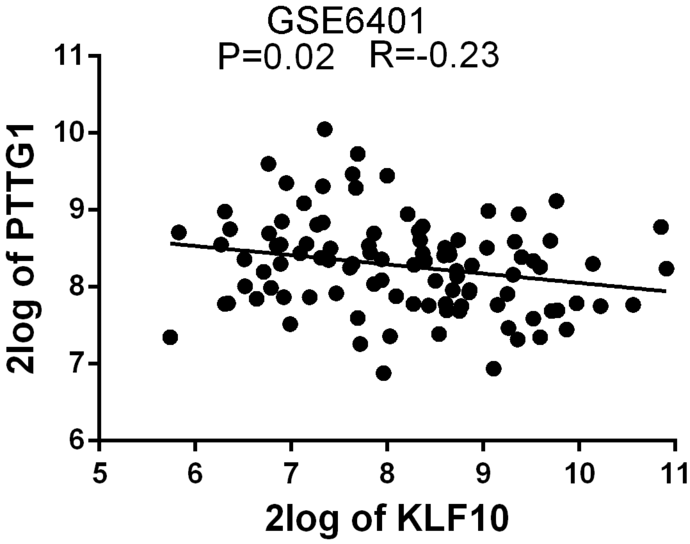

Figure S5 The GEO dataset (GSE6401) from R2: Genomics Analysis and Visualization Platform (http://r2.amc.nl) showed an inverse relationship between KLF10 and PTTG1.

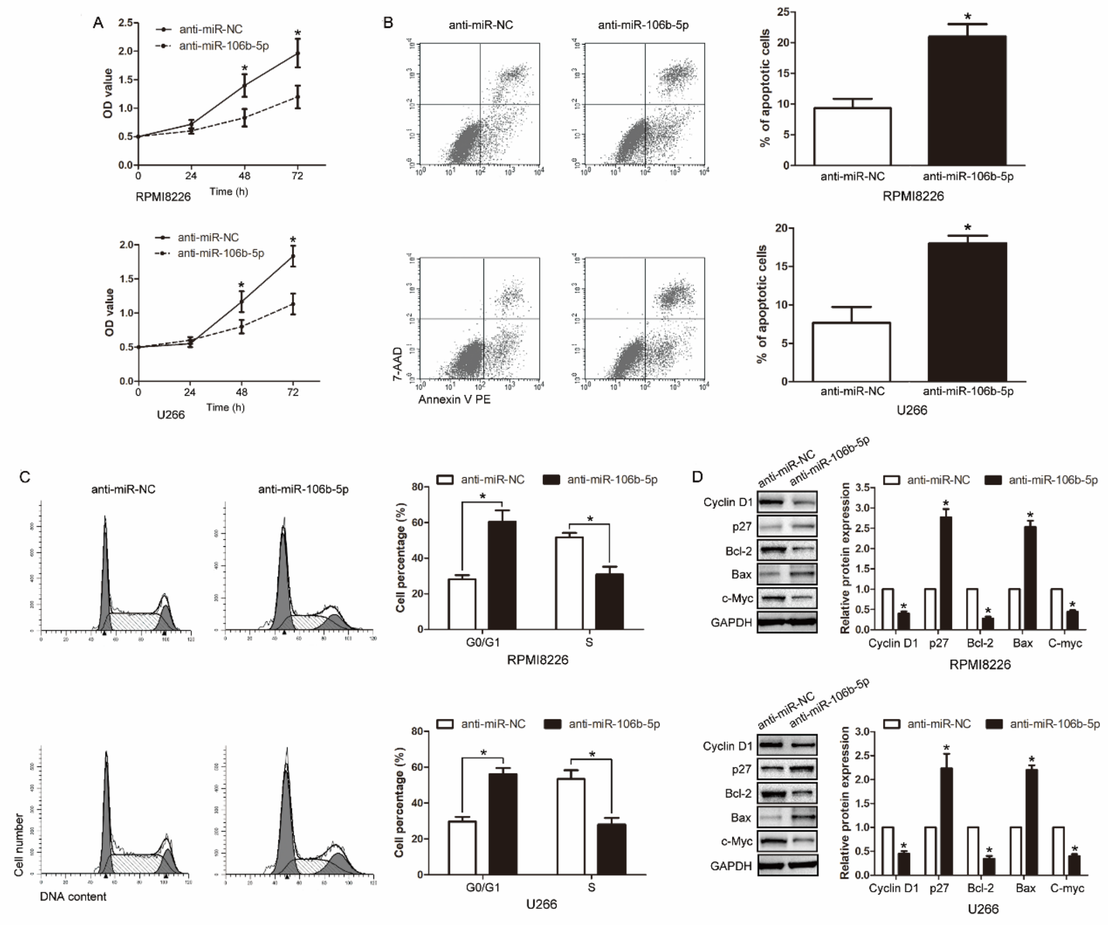

Figure S6. Inhibition of miR-106b-5p mimics KLF10-induced biological effects on MM. miR-106b-5p knockdown (A) suppressed cell proliferation (B) and promoted apoptosis (C) and inhibited cell cycle progression. (D) WB measured the cycle- and apoptosis-associated factors. *P<0.05.
